# PD-L1 in Systemic Immunity: Unraveling Its Contribution to PD-1/PD-L1 Blockade Immunotherapy

**DOI:** 10.3390/ijms21165918

**Published:** 2020-08-18

**Authors:** Ana Bocanegra, Ester Blanco, Gonzalo Fernandez-Hinojal, Hugo Arasanz, Luisa Chocarro, Miren Zuazo, Pilar Morente, Ruth Vera, David Escors, Grazyna Kochan

**Affiliations:** 1 Oncoimmunology Group, Biomedical Research Centre Navarrabiomed-UPNA, IdISNA, Irunlarrea 3, 31008 Pamplona, Spain; ester.blanco.palmeiro@navarra.es (E.B.); hugo.arasanz.esteban@navarra.es (H.A.); luisa.chocarro.deerauso@navarra.es (L.C.); miren.zuazo.ibarra@navarra.es (M.Z.); pilarmorente98@gmail.com (P.M.); descorsm@navarra.es (D.E.); 2 Department of Oncology, Complejo Hospitalario de Navarra, IdISNA, Irunlarrea 3, 31008 Pamplona, Spain; gonzalo.fernandez.hinojal@navarra.es (G.F.-H.); ruth.vera.garcia@navarra.es (R.V.)

**Keywords:** PD-L1, immunotherapy, immune, checkpoint inhibition, systemic myeloid subsets, liquid biopsy, biomarkers

## Abstract

The use of monoclonal antibodies targeting PD-1/PD-L1 axis completely changed anticancer treatment strategies. However, despite the significant improvement in overall survival and progression-free survival of patients undergoing these immunotherapy treatments, the only clinically accepted biomarker with some prediction capabilities for the outcome of the treatment is PD-L1 expression in tumor biopsies. Nevertheless, even when having PD-L1-positive tumors, numerous patients do not respond to these treatments. Considering the high cost of these therapies and the risk of immune-related adverse events during therapy, it is necessary to identify additional biomarkers that would facilitate stratifying patients in potential responders and non-responders before the start of immunotherapies. Here, we review the utility of PD-L1 expression not only in tumor cells but in immune system cells and their influence on the antitumor activity of immune cell subsets.

## 1. Introduction

Immune checkpoint inhibition (ICI) using monoclonal antibodies targeting the PD-1/PD-L1 axis are currently approved by the FDA for clinical use, with very good results in terms of improved progression-free survival (PFS) and overall survival (OS) in a variety of cancer types, including melanoma, lung, head and neck cancer and, invasive urothelial carcinoma. Nowadays, the management of the patients relies on PD-L1 tumor expression, the case of non-small cell lung cancer (NSCLC) being a particularly good instance. Anti-PD-1 (pembrolizumab, nivolumab) and anti-PD-L1 (atezolizumab) antibodies are administered to NSCLC patients as monotherapies after progression to a first-line treatment with conventional chemotherapy [1,2,3,4]. Standard first-line treatment, however, is still platinum-based chemotherapy or pembrolizumab (only for patients with a tumor PD-L1 expression higher than 50% [5]). The combination of PD-1/PD-L1 inhibitors plus chemotherapy received FDA approval in 2018 for first-line treatment of NSCLC patients, following favorable results in phase III clinical trials (KEYNOTE-021 study [6], IMpower150 study [7], KEYNOTE-189 study [8], KEYNOTE-407 study [9] and IMpower131 study [10]). The combination of ICIs is currently under evaluation (CheckMate-012 study [11]).

Despite their clinical success, anti-PD-1/PD-L1 immunotherapies do not render durable responses for all patients. Anti-PD-1/PD-L1 monotherapies achieve overall response rates (ORR) of around 20–30% in some tumor types such as melanoma or NSCLC. Furthermore, around 11–30% of patients develop immune related adverse events (irAEs) [12], such as pneumonitis, hypothyroidism, arthralgia, or vitiligo, as the result of ICI-induced T-cell hyperactivation and reduced immune tolerance towards healthy tissues [13].

**Figure 1:**
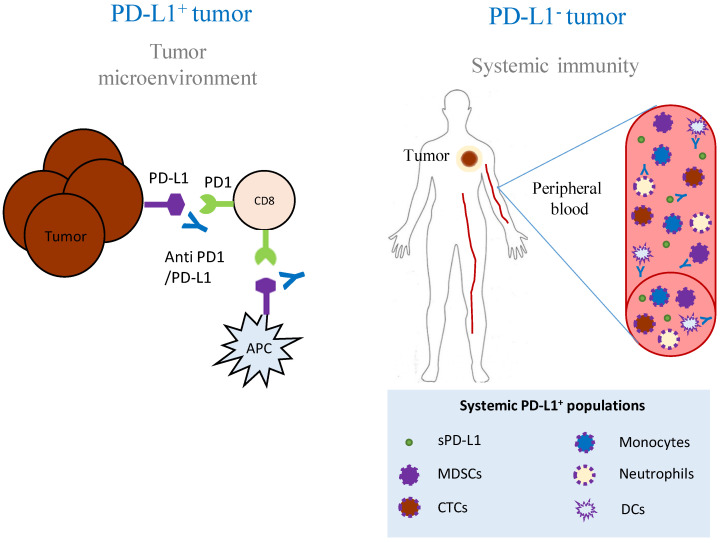
While clinical responses of cancer patients undergoing PD-1/PD-L1 blockade therapies may be explained by the suppression of the canonical PD-L1/PD-1 signaling axis, the fact that some patients with negative tumor PD-L1 expression still achieve objective responses highlights the contribution of PD-L1^+^ systemic immunity—particularly the myeloid compartment—to this kind of treatment. sPD-L1, soluble PD-L1; MDSC, myeloid derived suppressor cells; CTC, circulating tumor cell; DC, dendritic cell; APC, antigen presenting cell.

All these drawbacks, added to the high cost of these treatments, are evidences of the urgent need to develop a system to effectively stratify patients in order to restrict the candidates to those most likely to benefit from ICI. Although a very large body of research is devoted to the discovery of reliable biomarkers of response to ICI, the only clinically approved biomarker to date for patient selection for immunotherapy is PD-L1 expression in tumor cells measured by immunohistochemistry (IHC), along with *EGFR* and *BRAF* mutations and *ALK* and *ROS1* rearrangements [14]. However, PD-L1 as a biomarker shows limitations. Among them, there is no consensus about the antibody clones that might be employed, neither on the criteria to define PD-L1 positivity or even the most appropriate testing platforms. Other concerns to be taken into account are the dynamic and heterogeneous PD-L1 expression within tumors, which might differ between the biopsy and the rest of the tumor tissue, the time gap between the biopsy and therapeutic decisions, as well as the clinical evidences reporting cases of patients who are diagnosed as PD-L1 low or negative tumors and still respond to these treatments [15,16,17]. These facts support the possibility that other immune cell populations expressing PD-L1 are influencing clinical outcomes in PD-L1/PD-1 blockade immunotherapies (Figure 1).

Apart from tumor PD-L1 expression and the study of the immunosuppressive tumor microenvironment, liquid biopsy has emerged as a promising alternative for biomarker identification. The advantages offered by this technique include the availability of samples (only a small amount of blood is required), minimally invasive sample collections, real-time monitoring of treatment responses or resistances, and the suppression of the bias introduced by the spatial and temporal heterogeneity of the tumor. Conversely, this technique still lacks standardization and clinical validation. The research on liquid biopsy is increasing and has been widely reviewed elsewhere [16,18,19].

## 2. PD-L1 as a Tissue Biomarker. Problems and Limitations

PD-L1 expression by antigen-presenting cells (APCs) is a physiologically occurring mechanism to inhibit the activation of T lymphocytes, thus limiting the magnitude and duration of immune responses and preventing auto-immune reactivity. However, this mechanism is also utilized by tumors to escape from immune surveillance. Despite the usefulness of tumor PD-L1 testing by immunohistochemistry to enlarge the number of patients with a high probability of benefiting from ICIs, it faces some limitations. First, the use of different assays renders a plethora of cutoff values to define PD-L1 positivity. Actually, scoring algorithms used to assess PD-L1 positivity differ depending on the way staining patterns are interpreted. Then, the most frequently employed antibody clones for IHC are 22C3, 28-8, SP263, and SP142, and not all of them provide high concordance rates among different independent testing platforms [20]. This lack of standardization is highly related to the fact that the development of the different PD-L1 assessment techniques has been associated with the diverse clinical trials that have preceded the approval of particular anti-PD-1/PD-L1-targeted therapies. Thus, each method of PD-L1 detection has been developed by different pharmaceutical companies and the protocols and thresholds for positivity are associated with the methodology used in each trial. Nevertheless, considerable efforts are being made to harmonize the diverse methodologies due to the relevance of this biomarker in therapeutic decisions. A proper technical consensus would definitely increase the chances for a patient of receiving a favorable treatment or to be rejected an unhelpful therapy.

Collecting tumor biopsies for PD-L1 testing sometimes requires invasive procedures and histological samples may not always be available, particularly for some types of cancers such as advanced-stage NSCLC. Moreover, in the case of patients treated with immunotherapy as a second or further line of treatment, there is a time gap between the diagnosis and the clinical decisions during which intermediate treatments such as conventional chemotherapy, may alter PD-L1 expression in tumors. The dynamic regulation of PD-L1 expression could explain clinical cases showing that patients diagnosed as tumor PD-L1 negative show objective responses to atezolizumab (an anti-PD-L1 antibody) as a second-line treatment [21].

Another concern is the heterogeneous nature of tumor, that may affect the PD-L1 quantification depending on the origin of the biopsy (primary tumor or metastasis), the degree of intratumoral heterogeneity and the sampling methodology (biopsy or tumor resection) [22].

## 3. Systemic Biomarkers and PD-L1/PD-1 as Potential Systemic Biomarkers

Cancer research is predominantly dominated by the in-depth analysis of the tumor and the tumor microenvironment (TME). However, the tumor not only induces local immune dysfunction, but also distant immune changes that enable its proliferation and dissemination. Thus, cancer is also a systemic disease [23], and even tumor-targeted immunotherapies require systemic immune responses to be effective [24]. Therefore, the comprehensive view of the immune system in the context of the whole organism (the so-called “tumor organismal environment” [25]) should be considered. We will review hereafter the contribution of systemic elements not located in the TME but which can substantially affect tumor development and immunotherapy success, including soluble factors and circulating cell populations with a special focus on the role of PD-L1 as a major immune regulator (Table 1).

### 3.1. Soluble Serum Biomarkers

Most ICIs, including PD-1 and PD-L1 can be detected in two forms: attached to the membrane of tumor or immune cells (membrane-bound form, mPD-1/mPD-L1) and as soluble proteins in the plasma or serum (soluble forms, sPD-1/sPD-L1). The later are mainly generated by proteolitic cleavage of the membrane bound form by matrix metalloproteinases. Another source of sPD-L1 might be the alternative splicing of the PD-L1 mRNA. Tumor cells, T cells, myeloid cells, and the tumor microenvironment may be sources of sPD-L1 [35].

Several studies report an association between a high pre-treatment level of sPD-L1 in the plasma of cancer patients and a worsened clinical outcome after immunotherapy. Okuma et al. reported that 75% of NSCLC patients with an elevated baseline level of sPD-L1 showed progressive disease after treatment with nivolumab, with shorter time to treatment failure and reduced OS than patients with a low baseline level of sPD-L1 [26]. In agreement with this study, Meyo et al. reported that NSCLC patients with high baseline levels of sPD-1 and sPD-L1 rendered shorter PFS and OS after treatment with nivolumab [27]. Costantini et al. found no correlation between pre-treatment levels of sPD-L1 and response to nivolumab in NSCLC patients. However, they reported a significant elevation of sPD-L1 in non-responders at the moment of the first tumor evaluation under nivolumab, and higher ORR in patients with decreased or stable sPD-L1 concentrations from the start of immunotherapy to the first tumor evaluation [28]. Similarly, Ando et al. analyzed the evolution of sPD-L1 levels in the plasma of NSCLC and gastric cancer patients under anti-PD-1 treatment and reported an association between reduced sPD-L1 after 4 cycles of treatment and tumor regression [29]. In contrast, Chiarucci et al. reported a significant correlation between longer OS of mesothelioma patients treated with a combination of tremelimumab and durvalumab with low baseline sPD-L1 in sera, along with an increase in sPD-L1 from baseline to the first cycle of treatment [30]. Zhou et al. described that a rise in sPD-L1 after 5 months of treatment with pembrolizumab correlated with partial responses in a cohort of melanoma patients [31], suggesting that different mechanisms explaining the production of sPD-L1, or alternatively, its action is associated with different immune checkpoint inhibitors or different cancer types. Anyway, a general consensus exists on increased sPD-L1 in serum in cancer patients compared to healthy donors [30,31,36,37,38,39].

The sources of increased sPD-L1 and mechanisms behind its detrimental role over immunotherapy are not fully understood yet. Two hypotheses have been proposed [27]. First, sPD-L1 might bind to PD-1 on the surface of CD8 T lymphocytes providing an inhibitory signal, thus suppressing their cytotoxic activity and contributing to tumor immune evasion. Interestingly, the presence of sPD-1 would confer a pro-immunologic role to this molecule, since its binding to the membrane-associated PD-L1 on tumor or APCs would act as a PD-L1 blocking agent, thus preventing the inhibition of T cells through the PD-1/PD-L1 axis. On the other hand, these soluble molecules could act as decoys for anti-PD-1/PD-L1 antibodies, with a potential capacity to reduce their pharmacodynamic activity by impeding their checkpoint blockade function. A balance between these two possible mechanisms could contribute to their effects on the clinical activities of ICIs. In any case, the fact that sPD-L1 has a detrimental role in the clinical outcome of melanoma patients treated with anti-CTLA4 [31] antibodies strongly suggests the existence of other mechanisms apart from a direct interaction between the soluble and membrane-bound forms in the context of ICI immunotherapy.

Some studies have identified tumor cells as an important source of sPD-L1 and have associated increased levels with a larger tumor mass. Therefore, the increase in sPD-L1 may not have a direct detrimental role, but the worsened clinical outcomes would only reflect the tumor progression [28,38]. Other authors argue that the lack of correlation between tumor PD-L1 (determined by immunohistochemical analysis) and sPD-L1 indicates that different immune cells may be upregulating PD-L1 expression as a response to pro-inflammatory cytokines such as IFNγ and IL6, in the context of ICI immunotherapy [37]. In any case, the result would be an immunosuppressive status since sPD-L1 would retain its receptor-binding capacity and could induce T cell inhibition, thus impairing systemic host immunity [40].

Although easily quantifiable, the use of sPD-L1 as a predictive biomarker of response to immunotherapy is under debate because of the absence of standardized methods and cut-off values. In addition, several common disorders and physiological conditions alter the plasma level of sPD-L1, such as inflammation [41], allergies [42], auto-immune [43] and infectious diseases [44], diabetes [45], aging [46], and pregnancy [47].

### 3.2. PD-L1 Expression on Circulating Tumor Cells

Circulating tumor cells (CTCs) have an important role in metastasis. They arise from solid tumors and while the majority of them die in circulation, with an averaged survival in circulation of 2.5 h, a proportion of them can seed on distant organs leading to the establishment of metastases. CTCs are difficult to detect and isolate as a consequence of their low frequency in peripheral blood (less than 1 CTC/mL) [48,49,50].

Currently, a variety of strategies to isolate and analyze CTCs have been developed [51]. They represent an attractive alternative to tumor tissue biopsies since the techniques for isolation and purification are poorly invasive with only a small volume of blood sample required. It has been reported that CTC clusters of more than two or three cells have higher metastatic potential than single CTCs, although they have a shorter survival in blood [52].

It is difficult to compare the expression of PD-L1 in CTCs with the corresponding expression in cancer cells from the tumor microenvironment. This is a direct reflection of the heterogeneous PD-L1 expression in the whole tumor mass as well as the lack of immunomodulatory signals from the tumor microenvironment once CTCs are in circulation. Therefore, the correlation between CTC PD-L1 expression and PD-L1 expression in the tumor still remains controversial [53,54]. A growing number of studies are highlighting the relevant value of this marker as a predictive tool for immunotherapy response. Guibert et al. reported a correlation between baseline PD-L1^+^ CTCs and progressive disease in NSCLC patients [32]. Similarly, Nicolazzo et al. demonstrated that the presence of PD-L1^+^ CTCs at baseline and after 3 and 6 months of treatment with nivolumab correlated with progression and a worsened clinical outcome of NSCLC, suggesting that the persistence of this population might be a mechanism of resistance [33].

### 3.3. PD-L1 Expression on Systemic Myeloid Populations

PD-L1 expression is a mechanism commonly used by proliferating tumor cells to evade immune rejection. The interaction between tumor PD-L1 and its receptors, PD-1 and CD80 on the surface of cytotoxic T cells, is responsible for the neutralization of anti-tumor immune responses. That makes the PD-1/PD-L1 axis a major therapeutic target for immune checkpoint blockade-based immunotherapy. Strikingly, patients with a low expression of tumor PD-L1 still respond to anti-PD-L1 blockade treatments, thus evidencing that the expression of this immune checkpoint by immune cells (mainly myeloid cells [21]) may be key in determining clinical outcomes [55] (Table 2).

#### 3.3.1. Monocytes

Monocytes are circulating myeloid cells that upon recruitment to sites of inflammation, secrete pro-inflammatory cytokines, and differentiate into macrophages and dendritic cells. In humans, monocytes are classified into three main subtypes: classical “reparative” (CM; CD14^+^ CD16^−^), intermediate “inflammatory” (IM; CD14^+^ CD16^+^), and nonclassical or “patrolling” (NCM; CD14^−^ CD16^+^) monocytes. Circulating CM can undergo apoptosis or differentiate into NCM through an intermediate stage (IM) into the bloodstream and other organs. During differentiation, CM progressively lose CD14 expression and acquire CD16. NCM patrol the vasculature via a mechanism that requires the fractalkine receptor CX3CR1, engulfing apoptotic endothelial cells and sensing danger signals [69]. While lymphocytes are the predominant cells in PBMCs of NSCLC patients, monocytes and macrophages are the most frequent populations of the myeloid fraction in peripheral blood [70].

In a murine model, Bharat et al. demonstrated NCM to be involved in the recruitment of neutrophils through the production of chemokines such as CCL2 [69]. A beneficial role of NCM depletion has been associated with clinical benefits in a variety of diseases, including arthritis, traumatic brain injury, and cardiac failure, due to reduced inflammatory-associated tissue injury. In recent years, the contribution of NCM subsets to cancer biology is being increasingly investigated. While CM promotes tumorigenesis and metastasis, NCM are actively recruited to the lungs in a CX3CR1-dependent way and interact with metastasizing tumor cells, gather tumor cell debris from the lung vasculature and recruit and activate natural killer cells (NKs). These effects control the hematogenic spread of tumor to the lungs [71]. In fact, several studies reported a correlation between CX3CL1 production by tumor cells or tumor-associated cells and a good prognosis [72,73]. In contrast, the presence of NCM within the tumor and the subsequent recruitment of neutrophils driven by them, leads to the suppression of T cell-mediated anti-tumor immunity due to the release of immunosuppressive IL10 in a mouse model of colorectal cancer [74]. These results indicated that NCM subsets possess divergent roles in the context of anti-cancer immunity.

The frequency of circulating IM has been associated with a worsened cancer prognosis [75,76]. However, melanoma patients with higher baseline NCM percentages responded to ipilimumab treatment due to the involvement of these monocytes in T_reg_ depletion via antibody-dependent cell-mediated cytotoxicity [77]. The baseline frequency of circulating CM has also been reported to be a strong predictive biomarker of response to anti-PD-1 immunotherapy in melanoma patients [78].

PD-L1 together with the chemokine receptor CX3CR1 has recently been reported to be a marker of NCM in peripheral blood and bone marrow under inflammatory conditions that promote T cell survival in tertiary lymphoid organs [57]. Monocytes in chronic lymphocytic leukemia acquire a PD-L1^+^ phenotype through the transfer of non-coding RNAs via tumor-derived extracellular vesicles, thus triggering local and systemic pro-tumorigenic functions [56]. In agreement with this, the depletion of circulating monocytes in leukemia patients enhanced NK activation. This result evidenced an immune evasion strategy driven by PD-L1^+^ inhibitory CD163^+^ monocytes promoting exhaustion of PD-1^high^ NK cells [58].

#### 3.3.2. Macrophages

Macrophages are myeloid cells that differentiate from circulating monocytes. Cells from this myeloid subset are commonly classified as M1 pro-inflammatory macrophages and M2 immunosuppressive macrophages. While M1 macrophages play a key role in antigen presentation and pro-inflammatory cytokine production, the M2 macrophages mainly release anti-inflammatory cytokines and contribute to anti-inflammatory processes such as wound healing. The M1 phenotype is characteristic of the onset of immune responses, whereas the M2 polarization occurs at the resolution stage of inflammation. Cytokines such as IFNγ and GM-CSF drive monocyte differentiation towards M1 macrophages, while M-CSF, IL-4, IL-13, and IL-10 are responsible for M2 differentiation. Once recruited to the tumor microenvironment in a CCL2-dependent manner, macrophages differentiate into tumor-associated macrophages (TAMs). However, the phenotype of macrophages is dynamic and reversible depending on the surrounding cytokines, being M2 the major phenotype of TAMs.

A significant body of research has focused on TAMs due to their influence on the outcome of cancer [79]. TAMs support tumor cell invasion and intravasation at primary tumor sites, enhance angiogenesis, promote survival, extravasation, and growth of metastasizing tumor cells. However, TAMs do play a dual role within the tumor microenvironment. In fact, PD-L1 on the surface of tumor cells can enhance phagocytic capacity of PD-1 expressing TAMs [80], suggesting a positive contribution of this population in clinical outcomes of PD-1/PD-L1 blockade strategies. In agreement with this, Dhupkar et al. reported regression of osteosarcoma lung metastasis by an anti-PD-1-mediated mechanism which was dependent on effector M1 macrophages [81].

Nevertheless, TAMs are not the unique macrophage population with metastasis-promoting activity. Another distinct macrophage population called metastasis-associated macrophages (MAMs) originate from monocytes and myeloid precursors by an M-CSF dependent differentiation process. These cells are chemoattracted by circulating tumor cells (CTCs)-derived CCL2 into the metastatic organ [82]. MAMs express higher levels of PD-L1 than monocytes, although they suppress effector T cell activity by a ROS-dependent but checkpoint receptor-independent mechanism [59].

#### 3.3.3. Dendritic Cells

Dendritic cells (DCs) are professional antigen-presenting cells (APCs) distributed throughout the whole body. Two major populations of dendritic cells can be found in the peripheral blood: myeloid CD11c^+^ DCs (mDCs) and plasmacytoid DCs (pDCs). The numbers of peripheral blood pDCs in NSCLC patients increase in advanced stages of the disease [83].

Anti-PD-1/PD-L1 immunotherapies rely on PD-1/PD-L1 blockade between T cells and tumor cells to overcome tumor-induced T-cell immune suppression. However, PD-L1 is highly expressed by many myeloid cells, including antigen-presenting cells such as dendritic cells (DCs). While PD-1 binds to two possible ligands (PD-L1 and PD-L2), PD-L1 interacts with PD-1 and CD80. In addition to the main mechanism of action, the clinical efficacy of anti-PD-L1 treatments has also been associated with blockade of the *cis* interaction between PD-L1 and CD80 on DCs, thus enabling the interaction of CD80 with CD28 on T cells [60,61]. This mechanism would apply to peripheral and tumor-associated DCs, and could explain the reinvigoration of anti-tumor CD8 T cell responses triggered by immunotherapy.

The crosstalk between DCs with other immune cells mediated by PD-1/PD-L1 interactions is quite diverse. Ray et al. reported that DCs may abrogate cytotoxic NK action through PD-L1-PD-1 ligation, that can be recovered after anti-PD-L1 treatment [62]. DCs also induce T_reg_ expansion in a PD-L1 dependent mechanism in a murine model [63], suggesting that this immunosuppressive population might also be targeted by anti-PD-L1 immunotherapy. Moreover, the co-culture of mature DCs with cytokine-induced killer cells (CIKs, a population of CD56^+^ CD3^+^ cells with the ability to kill cancer cells in an MHC-unrestricted manner) in the presence of pembrolizumab showed an increased proliferation and cytotoxic capacity of this population in a liver cancer model in vitro and in vivo [64].

#### 3.3.4. Myeloid-Derived Suppressor Cells (MDSCs)

MDSCs are a heterogeneous plastic myeloid population with strong immunosuppressive activities that promote tumor angiogenesis and metastasis. Since 1970, MDSCs have been reported to be involved in the development of different types of tumors and in chronic inflammation through a variety of mechanisms, including oxidative stress and nutrient depletion via inducible nitric oxide synthase (iNOS) and arginase production [84]. The high expression of PD-L1 in MDSCs constitutes an important element for their immunosuppressive activities [85]. As a result of the interaction, they inhibit T cell effector activity.

MDSC are currently divided into two subsets: monocytic MDSC (mMDSC) and granulocytic (or polimorphonuclear PMN) MDSC (gMDSC). In mice, monocytic MDSC have a phenotype of CD11b^+^ Ly6C^+^ Ly6G^−^ CD11C^−^ F4/80^−^, while granulocytic MDSCs have a phenotype of CD11b^+^ Ly6C^−^ Ly6G^+^ CD11C^−^ F4/80^−^ [84,86,87].

In humans, monocytic MDSCs are characterized as CD11b^+^ CD33^+^ CD14^+^ CD15^−^ HLA-DR^−/lo^ and CD11b^+^ CD14^−^ CD15^+^ and CD66b^+^ HLA-DR^−^ for granulocytic MDSCs. Additionally, early stage MDSCs are distinguished by a phenotype Lin- CD11b^+^ CD33^+^ HLA-DR^−^ [88].

Circulating MDSCs in healthy individuals and mice are virtually undetectable or at very low numbers. In cancer patients as well as in other pathological conditions such as sepsis and chronic infectious diseases, MDSCs accumulate as a result of chronic inflammation and exert their immunosuppressive activity towards innate and adaptive immunity [89]. The correlation of MDSC numbers with cancer stage and tumor burden in different types of cancer has been studied [90,91]. Indeed, elevated MDSC frequencies in circulation are associated with poor outcomes in patients with solid tumors [92,93]. Different studies have correlated the elevation of circulating MDSC numbers with poor clinical responses to anti-cytotoxic T lymphocyte antigen-4 (CTLA-4) and anti-PD-1 immunotherapies in advanced melanoma patients [65,66,93]. MDSCs and TAM likely constitute the major myeloid populations expressing PD-L1, and some strategies are based on their elimination to reduce their T cell suppressive activities [67].

#### 3.3.5. Granulocytes/Neutrophils

Neutrophils are the most abundant leukocytes in circulation and constitute the primary response from cells of the innate immune system. For a long time, the role of neutrophils has been underestimated because of their short lifetime. However, their lifespan can change by proinflammatory factors such as IFN-γ [94]. The influence of proinflammatory factors causes neutrophil polarization, enhancing their tumor infiltration and their pro- or antitumor activity [95]. Neutrophils can have significant anti-tumor activity [96] Expression of CXCL1, CXCL2, and CXCL5 chemokine driven by hypoxia within the tumor microenvironment cause neutrophil recruitment to the tumor site. Tumor infiltration with neutrophils has been associated with higher overall survival in CRC patients [97]. In contrast, the increase of neutrophil infiltration has also been associated with anti-PD-1 failure in NSCLC patients [98]. The effects of different stimuli over neutrophil differentiation and function are still under investigation due to their plasticity. Yoshimura and Takahashi have shown that the influence of IFN-γ induces PD-L1 upregulation in neutrophils, which mediates inhibition of T cell proliferation [94]. Cheng and collaborators showed that IL-6 secreted by cancer-associated fibroblasts induce PD-L1 upregulation on neutrophils [99]. Kleijn and colleagues showed that inflammation enabled the suppression of lymphocyte proliferation associated with PD-L1 up-regulation in systemic neutrophils [68]. Castell identified in a murine model different neutrophil function over CD4 suppression [100]. In another study, the neutrophil-to-lymphocyte (NLR) ratio has been correlated with poor prognosis in different cancers [101,102]. Although PD-L1 expression in these neutrophils was not studied, we can speculate that an increase in NLR could be associated with up-regulation of PD-L1 expressing neutrophils.

### 3.4. PD-L1 Expression on Systemic Lymphoid Populations

In the context of the tumor microenvironment, Diskin et al. reported a pleiotropic role of T cells-expressed PD-L1 over the innate and adaptive immune system. They described a mechanism through which PD-L1 on tumor-infiltrating lymphocytes (TILs) transmits forward and backward signals to regulate immune responses in the tumor. Thus, the tolerogenic role of PD-L1 would compete with the inhibitory action of PD-1, suppressing neighbor T cells even in the absence of PD-L1^+^ myeloid cells, and inducing M2-like reprogramming of TAMs. As a global effect, PD-L1 expressing T lymphocytes would promote tumor growth and intratumoral immune tolerance [103,104].

Apart from TILs, PD-L1 expression by circulating CD4 and CD8 T cells has also been reported to be associated with the clinical outcome of cancer patients undergoing immunotherapy. Jackelot et al. reported that PD-L1 expression on peripheral T cells rendered prolonged OS and PFS in melanoma patients treated with ipilimumab, as well as a lack of relapse with ipilimumab + nivolumab combination therapy in patients with PD-L1^+^ circulating CD8 T lymphocytes [34]. Although the authors propose the expression of PD-L1 on circulating T cells as a predictive biomarker of response to anti-CTLA4 immunotherapy, it has been argued by Brochez et al. that the correlation of PD-L1^+^ lymphocytes with the clinical outcome is mainly related to the existence of a negative immune context characterized by the presence of MDSCs and T_reg_, and decreased pDCs, rather than to the contribution of that particular population to the effect of immunotherapy [105].

## 4. Conclusions

Considering all the studies published by numerous authors in murine models as well as in clinical studies, PD-L1 expression on different non-tumor cell types, including several immune cell subsets, conditions the availability of T cells with effector activities that can respond to stimulation by ICIs. To make feasible the use of PD-L1 on immune cells and/or soluble proteins as a biomarker, systematic studies and correlations with objective responses in larger patient cohorts are required. These studies could unravel the role of PD-L1 expression particularly in myeloid cell types, and the implications of PD-L1 blockade in these cells over clinical responses. The fact that PD-L1 expression is intrinsically dynamic and generalized to a wide range of immune cells means that therapeutic decisions concerning patient access to anti-PD-1/PD-L1 treatments based on tumor PD-L1 expression exclusively are quite limited since other immune contributions are being ignored that could have an important contribution to predicting clinical responses to these treatments. This highlights the relevance of identifying the role of systemic PD-L1 assessment as a biomarker of response in ICI. Furthermore, although PD-L1 testing usually enables the enlargement of patient cohorts that benefit from immune checkpoint blockade, it does not apply to every tumor type, such as melanoma. Not only is the kind of tumor involved in predicting immunotherapy response, but also other factors such as sex. Thus, male patients show higher response rates to ICIs compared to female patients, while the opposite tendency may apply to the combination of ICIs and chemotherapy. Sex differences are also observed in the efficacy of certain biomarkers of response to immunotherapy, such as tumor mutation burden.

Taken all together, the tendency moves towards a combination of PD-L1 testing with other emerging and/or well-established biomarkers (tumor mutation burden, neoantigenic signature, DNA mismatch repair, etc.) in a tumor-specific setting. The coming challenge will be to integrate the wide plethora of available biomarkers with the potential to predict responses to ICI immunotherapy under the form of clinically useful algorithms for better patient management.

## Figures and Tables

**Table 1:** Summary of studies on PD-L1 beyond the tumor microenvironment.

Cancer Type	PD-L1 Source	Number of Patients/Treatment	Main Results	References
NSCLC	Soluble PD-L1	39 patients/nivolumab	Elevated baseline levels of sPD-L1 correlate with progressive disease	Okuma et al. [26]
NSCLC	Soluble PD-L1	51 patients/nivolumab	High baseline levels of sPD-1 and sPD-L1 render shorter PFS and OS	Meyo et al. [27]
NSCLC	Soluble PD-L1	43 patients/nivolumab	Increased sPD-L1 in non-responders at first tumor evaluation	Costantini et al. [28]
NSCLC/gastric cancer	Soluble PD-L1	21 patients/nivolumab, pembrolizumab	Reduced sPD-L1 levels after treatment correlates with tumor regression	Ando et al. [29]
mesothelioma	Soluble PD-L1	40 patients/durvalumab + tremelimumab	Low baseline levels of sPD-L1 render longer OS	Chiarucci et al. [30]
Melanoma	Soluble PD-L1	100 patients/ipilimumab, bevacizumab, pembrolizumab	Increased sPD-L1 after treatment correlates with partial responses	Zhou et al. [31]
NSCLC	Circulating tumor cells	96 patients/nivolumab	High baseline CTC numbers associate with progression	Guibert et al. [32]
NSCLC	Circulating tumor cells	24 patients/nivolumab	Presence of PD-L1^+^ CTCs both at baseline and after treatment correlates with progression and worsened outcome	Nicolazzo et al. [33]
NSCLC	CD11b^+^ cells	32 patients/nivolumab, pembrolizumab, atezolizumab	Circulating PD-L1^+^ myeloid populations correlate with response to anti PD-L1/anti PD-1 treatment in NSCLC patients, independently of tumor PD-L1 expression	Bocanegra et al. [21]
Melanoma	Peripheral T cells	190 patients/ipilimumab, nivolumab	PD-L1 expression on peripheral T cells is a prognostic biomarker of OS and PFS	Jacquelot et al. [34]

**Table 2:** Summary of studies on PD-L1 in peripheral myeloid populations.

Population	Main Results	References
Monocytes	Monocytes acquire PD-L1^+^ phenotype via tumor-derived extracellular vesicles and exert pro-tumorigenic functions	Haderk et al. [56]
Non-classical monocytes (NCM)	PD-L1 is a marker of NCMunder inflammatory conditions and promotes T cell survival	Bianchini et al. [57]
Monocytes	PD-L1^+^ circulating monocytes promote exhaustion of PD-1^high^ natural killer cells	Vari et al. [58]
Metastasis associated macrophages (MAMs)	Despite PD-L1 expression, they suppress T cell activity by a ROS-dependent but checkpoint-independent mechanism	Kitamura et al. [59]
Dendritic cells (DCs)	The immunotherapy-driven blockade of the *cis* interaction of PD-L1 with CD80 on DCs enables the interaction CD80-CD28, thus reinvigorating cytotoxic CD8 T cell responses	Sigiura et al. [60], Mayoux et al. [61]
Dendritic cells (DCs)	PD-L1 blockade reverses natural killer cells suppression lead by DCs	Ray et al. [62]
Dendritic cells (DCs)	DCs induce the expansion of T_reg_ in a PD-L1 dependent mechanism	Liu et al. [63]
Dendritic cells (DCs)	PD-1 blockade induces proliferation and cytotoxic capacity of cytokine-induced killer cells co-cultured with DCs in a liver cancel model in vitro and in vivo, rendering enhanced clinical benefits	Zhang et al. [64]
Myeloid-derived suppressor cells (MDSCs)	High numbers of MDSCs were associated with poor survival in ipilimumab-refractory melanoma patients treated with nivolumab	Weber et al. [65]
Myeloid-derived suppressor cells (MDSCs)	PD-L1^+^ MDSCs are less frequent in peripheral blood as compared to tumor tissues. pSTAT1-IRF1 axis regulates PD-L1 expression in MDSCs	Lu et al. [66]
Myeloid-derived suppressor cells (MDSCs)	MDSC inhibition augments general and tumor-specific immunity in head and neck squamous cell carcinoma (HNSCC)patients	Califano et al. [67]
Neutrophils	IFNγ-induced expression of PD-L1 on circulating neutrophils suppress lymphocyte proliferation	De Kleijn et al. [68]

## Abbreviations

PD-1	Programmed death 1
PD-L1	Programmed death ligand 1
ICI	Immune checkpoint inhibition
FDA	Food and drug administration
PFS	progression-free survival
OS	overall survival
NSCLC	non-small cell lung cancer
ORR	overall response rates
irAEs	immune related adverse events
IHC	immunohistochemistry
APC	antigen presenting cells
TME	tumor microenvironment
CTC	circulating tumor cells
CTLA4	cytotoxic T-lymphocyte-associated protein 4
DC	Dendritic cells
IFNγ	interferon gamma
IL6	interleukin 6
sPD-L1	soluble PD-L1
NCM	nonclassical monocytes
IM	intermediate monocytes
CM	classical monocytes
CCL2	C-C Motif Chemokine Ligand 2
NK	natural killer cells
IL10	interleukin 10
CX3CR1	CX3C chemokine receptor 1
GM-CSF	granulocyte monocyte colony stimulating factor
M-CSF	macrophage colony stimulating factor
IL-4	interleukin 4
IL-13	interleukin 13
TAM	tumor-associated macrophages
MAM	metastasis associated macrophages
ROS	reactive oxygen species
PD-L2	Programmed cell death ligand 2
T_reg_	regulatory T cells
CIKs	cytokine-induced killer cells
MHC	Major histocompatibility complex
MDSCs	myeloid derived suppressor cells
iNOS	inducible nitric oxide synthase
mMDSCs	monocytic MDSCs
gMDSCs	granulocytic MDSCs
PMN	polymorphonuclear cells
HLA-DR	human leucocyte antigen DR
CRC	colorectal cancer
NLR	neutrophil-to-lymphocyte ratio
TIL	tumor infiltrating lymphocytes
sPD-L1	soluble PD-L1
